# Early Cytomegalovirus Reactivation in Renal Recipients Is Associated with High Levels of B Cell Maturation Antigen Transcript Expression Prior to Transplantation

**DOI:** 10.3390/ijms241310491

**Published:** 2023-06-22

**Authors:** Rafael Alfaro, Luis Rodríguez-Aguilar, Santiago Llorente, Victor Jimenez-Coll, Helios Martínez-Banaclocha, José Antonio Galián, Carmen Botella, María Rosa Moya-Quiles, Manuel Muro-Perez, Alfredo Minguela, Isabel Legaz, Manuel Muro

**Affiliations:** 1 Immunology Service, Hospital Clinico Universitario Virgen de la Arrixaca (HCUVA), Biomedical Research Institute of Murcia (IMIB), 30120 Murcia, Spain; raf.hellin@gmail.com (R.A.); diegoarmandogalian@hotmail.com (J.A.G.); rosa.moya2@carm.es (M.R.M.-Q.);; 2 Nephrology Services, Hospital Clinico Universitario Virgen de la Arrixaca (HCUVA), Biomedical Research Institute of Murcia (IMIB), 30120 Murcia, Spain; 3 Department of Legal and Forensic Medicine, Biomedical Research Institute of Murcia (IMIB), Regional Campus of International Excellence “Campus Mare Nostrum”, Faculty of Medicine, University of Murcia, 30100 Murcia, Spain

**Keywords:** BCMA, CMV, BAFF gene expression, acute rejection, kidney transplant, plasmablast

## Abstract

Cytomegalovirus (CMV) infection is the most frequent infection episode in kidney transplant (KT) recipients. Reactivation usually occurs in the first three months after transplantation and is associated with higher cellular and/or antibody-mediated rejection rates and poorer graft performance. CMV induces the expression of BAFF (B-cell-activating factor, a cytokine involved in the homeostasis of B cells), which communicates signals for survival and growth to B cells and virus-specific plasma cells via the R-BAFF (BAFF receptor), TACI (the calcium modulator, the cyclophilin ligand interactor), and BCMA (B cell maturation antigen) receptors. These molecules of the BAFF system have also been suggested as biomarkers for the development of alloantibodies and graft dysfunction. This prospective study included 30 CMV-IgG seropositive KT recipients. The expression levels of the genes BAFF-R, transmembrane activator and CAML interactor (TACI), and B cell maturation antigen (BCMA) in peripheral blood leukocytes (PBL) pre-KT were determined using qPCR. qPCR was also used to monitor CMV reactivation in the first three months following KT. The remainder of the KT recipients were classified as CMV− reactivation, and those with more than 500 copies/mL in at least one sample were classified as CMV+ reactivation. There were no discernible variations in the BAFF-R and TACI transcript expression levels. In the CMV+ group, we examined the relationship between the transcript levels and peak viremia. Peak viremia levels and BCMA transcript levels showed a strong correlation. BAFF-R and TACI expressions showed no measurable differences. In patients with early CMV reactivation, high BCMA receptor expression was associated with increased plasmablast, lymphocyte B cell class-switched levels (LBCS), and viral load. Our findings demonstrate that pre-KT BCMA transcript levels increased in KT recipients with early CMV reactivation. These transcript levels positively correlate with peak viremia and weakly with plasmablast and LBCS levels in PBLs.

## 1. Introduction

Cytomegalovirus (CMV) infection is kidney transplant recipients’ most frequent side effect. Reactivation typically takes place in the first few months following transplantation, and it has been linked to rising allograft rejection rates and impaired graft function [1].

Furthermore, the B-cell-activating factor (BAFF) is a cytokine involved in B cell homeostasis and is required to maintain healthy immunity. Immunodeficiencies result from a lack of BAFF, which prevents B cells from producing immunoglobulins (Igs).

The BAFF receptor (BAFF-R), the transmembrane activator and calcium modulator, the transmembrane activator and calcium modulator and cyclophilin ligand interactor (TACI), and the B cell maturation antigen are the three receptors that BAFF binds to in order to function (BCMA). Increased levels of BAFF cause abnormally high antibody (Ab) production and it is a survival factor for B cells that controls B cell maturation. BAFF levels are detected in patients with B-cell-mediated autoimmune diseases (systemic lupus erythematosus (SLE) and rheumatoid arthritis (RA)). BAFF is also found in kidney transplantation (KT) biopsies with acute rejection (AR) and correlates with Cd4+. Increased levels of BAFF may initiate alloreactive B/T cell immunity and thus promote AR [2]. Lower BAFF level transcripts, or higher levels of soluble BAFF (sBAFF), show a higher risk of producing DSAs, which bind with high affinity to the vascular endothelium of grafts and activate complement, resulting in neutrophil infiltration, fibrin deposition, and platelet aggregation [2,3]. BAFF is considered to be a prime therapeutic target. Targeting BAFF-R interactions may provide new therapeutic possibilities in transplant.

The TNF family cytokines, BAFF, and the proliferation-inducing ligand (APRIL) have a homotrimeric type II transmembrane structure [4]. BAFF and APRIL soluble forms are produced through the proteolytic processing of the membrane forms by the protease furin in consensus sequences. The membrane form of APRIL is efficiently processed in its soluble form, while BAFF can also be found in its membrane-bound form.

BAFF and APRIL can bind to BCMA and the TACI receptor. Furthermore, BAFF can also bind to R-BAFF [5]. BAFF and APRIL receptors are mainly expressed in B cells. BAFF-R is only weakly expressed in B cells at the late transition stage, but as the cells mature, its expression gradually increases [6]. When B cells enter the germinal center, their expression declines. They are re-expressed in memory B cells but not in plasmablasts or plasma cells.

Memory B cells, plasma cells, and a small population of activated CD27-negative B cells express the human inducible receptor known as TACI [7]. Only plasmablasts, plasma cells, and germinal center B cells can express BCMA. Apart from B cells, other cell types have observed the BAFF system molecules’ expression. For instance, BAFF-R, which Tregs constitutively express, has increased T cells’ expression upon activation. Nevertheless, in addition to B cells, monocytes and dendritic cells also express TACI [8].

BAFF promotes transitional B-lymphocyte maturation and mature B cell survival, while BAFF and APRIL promote plasma cell survival. In addition, memory B cells’ survival and reactivation are independent of BAFF- and APRIL-mediated signaling [9].

The BAFF receptors can separate from the membrane and function as soluble forms of BAFF and APRIL molecules. Soluble TACI (sTACI) antagonizes with BAFF and APRIL molecules, while soluble BCMA (sBCMA) only antagonizes with the APRIL molecule, thus preventing its binding to membrane-bound forms. BAFF-R is not released constitutively, requiring stimulation with BAFF and TACI co-expression. Soluble receptors can act as a negative feedback mechanism, blocking the action of BAFF and APRIL, thus inhibiting B-cell-mediated immune responses [7,10].

There are not many studies on soluble receptors in the field of KT, but they could be helpful as biomarkers of antibody-mediated responses to grafting [7].

The molecules of the BAFF system have been suggested as biomarkers for the development of alloantibodies and kidney graft dysfunction. Researchers hypothesized that because these patients’ post-KT BAFF levels were higher than those of control recipients, there may have been a dysregulation in the microenvironment that favored the development and activation of allo-reactive B cells. Concerning the gene expression of immune molecules in transplant, most studies in KT have focused on the study of mRNA from peripheral blood leucocytes (PBLs), kidney biopsies, or urine samples. The quantification of RNA allows for indirectly measuring the levels of gene expression in an individual [2].

Finally, CMV induces the expression of the BAFF molecule, which communicates signals for survival and growth to B cells and virus-specific plasma cells via the R-BAFF, TACI, and BCMA receptors, as published in [11]. In early times after surgery and pre and post transplant, CMV infection may influence the different subtypes and stages of B cells and BAFF system molecules’ expression to produce alterations in graft function and homeostasis. In order to predict CMV reactivations in the early post-transplantation stages, it is crucial to understand how to affect CMV reactivation in kidney graft function, alloantibody production, BAFF system gene expression, and the various B cell subtypes implicated in antibody production.

To predict reactivations in the early post-transplant stages, the current investigation evaluated transcript levels of BAFF system receptors in peripheral blood leucocytes at pre and post transplant in CMV-IgG seropositive kidney graft recipients.

## 2. Results

For this study, the patients were divided into two groups based on CMV infection: with (CMV+) and without CMV infection (CMV−). Both groups were similar in age and sex, with no significant differences (*p* = 0.365 and *p* = 1.000, respectively) in the rest of the parameters (*p* > 0.05). The post-transplant reactivation in our study series was 66.7%.

Table 1 displays the demographic information and clinical characteristics of kidney graft recipients.

In the population analyzed, the main indications for kidney graft transplantation were glomerulonephritis (30%) and polycystic kidney disease (16.7%) (Table 1).

Concerning donor characteristics, all donors were cadaveric; the median donor age was 60.9 years and, for donor gender, 61% were female and 39% were male.

### Dynamic Changes in Gene Expression after Transplantation

First, we look at changes in the expression of genes related to the BAFF system from the pre- to the post-transplant period in kidney transplant recipients with CMV-negative or -positive status.

**Figure 1:**
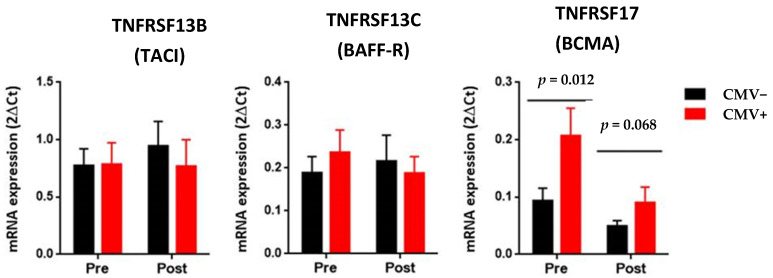
Comparison of the transcript levels of TNFRSF13C (BAFF-R), TNFRSF13B (TACI), and TNFRSF17 (BCMA) in peripheral blood from recipients of the CMV− group (*n* = 18) and recipients of the CMV+ group (*n* = 12). The amounts of mRNA were reported to the CD19 gene. The expression of the BAFF and APRIL genes was normalized using the HPRT gene as an endogenous reference, while the expression of the TNFRSF13C (BAFF-R), TNFRSF13B (TACI), and TNFRSF17 (BCMA) genes was normalized using the CD19 gene. Expression data are represented as the mean ± SEM. As previously published, statistical analyses were performed using the Wilcoxon test for related samples [2]. Values of *p* < 0.05 were considered significant.

According to this analysis, pre-transplantation (Figure 1, right graphic, *p* = 0.012) but not post-transplantation (*p* = 0.068) BCMA transcript expression was significantly up-regulated in the peripheral blood of kidney graft recipients in the CMV+ group compared to those in the CMV− group. Nonetheless, the BAFF-R (*TNFRSF13C*) and TACI (*TNFRSF13B*) genes’ transcript expression levels did not differ significantly from one another (*p* > 0.05, Figure 1, left and central graphics).

**Figure 2:**
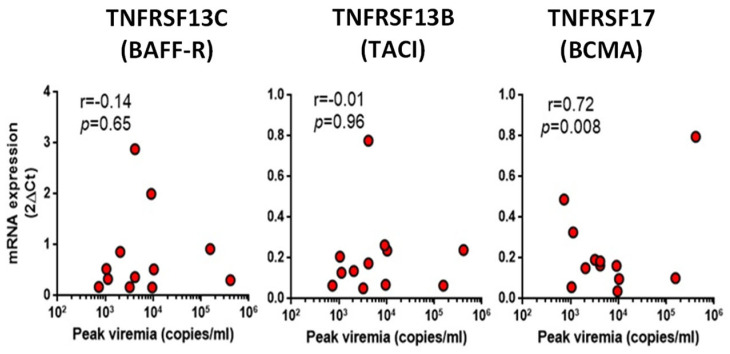
Peak viremia and transcript levels were correlated in the CMV+ group. Peak viremia and BCMA transcript levels were significantly correlated (r = 0.72, *p* = 0.008). There were no discernible differences between TNFRSF13C (BAFF-R) and TNFRSF13B (TACI). Correlation analyses were carried out using the Spearman index, as previously described [2]. The r corresponds to the correlation coefficient. Values of correlation and *p* values in pre- and post-transplantation Tx in all the analyzed BAFF molecule systems: BAFF—pre-Tx, r = 0.537, *p* = 0.06; post-Tx, r = 0.585, *p* = 0.07. APRIL—pre-Tx, r = 0.331, *p* = 0.21; post-Tx, r = 0.8, *p* = 0.07. BAFF-R—pre-Tx, r = 0.14, *p* = 0.65; post-Tx, r = −0.193, *p* = 0.54. TACI—pre-Tx, r = −0.01, *p* = 0.96; post-Tx, r = 0.126, *p* = 0.696. BCMA—pre-Tx, r = 0.72, *p* = 0.008; post-Tx, r = 0.416, *p* = 0.176. pre-Tx: pre-transplant; post-Tx: post-transplant.

Next, we also evaluated the correlation between transcripts levels of BAFF receptors and peak viremia in these patients of the CMV+ group. According to this analysis, peak viremia and BCMA transcript levels were significantly correlated (Figure 2, right graphic, r = 0.72, *p =* 0.008), but the possibility that data from a scattered individual may influence this statistic cannot be ruled out.

In this same analysis, no significant differences were observed in the other two BAFF receptors, the TNFRSF13C (BAFF-R) and TNFRSF13B (TACI) genes (*p* > 0.05, Figure 2, central and left graphics).

Finally, we analyzed different B cell populations concerning the CMV status of kidney graft recipients pre and post transplantation.

**Figure 3:**
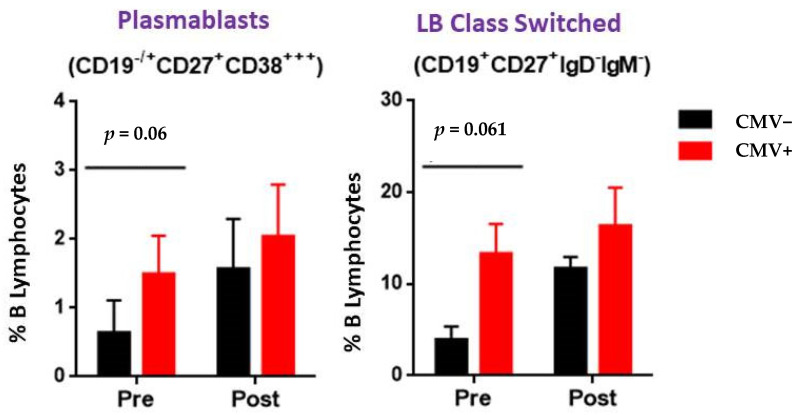
Kidney graft recipients suffering post-transplant CMV reactivation tended to have higher plasmablast and lymphocyte B class-switched numbers pre transplantation and were statistically borderline (*p* = 0.06 and *p* = 0.061, respectively) but not statistically significant (*p* > 0.05). Comparisons were made using the Mann–Whitney U test. Values are expressed as the mean ± SEM. *p* values < 0.05 were considered statistically significant for all analyses.

In this particular analysis, kidney graft recipients suffering post-transplant CMV reactivation tended to have higher plasmablasts (CD19^−/+^CD27^+^CD38**) and lymphocyte B class-switched (CD19^+^CD27^+^IgD^−^IgM^−^) numbers and percentages pre transplantation that were statistically borderline (*p* = 0.06 and *p* = 0.061, respectively) but not statistically significant (*p* > 0.05) (Figure 3).

## 3. Discussion

Studies of the gene expression of molecules of the BAFF system are scarce in kidney transplantation. However, studies have shown that the dysregulation of the expression of BAFF and some of its receptors could be associated with adverse events in the transplanted recipient, such as chronic graft dysfunction or apparition and the development of DSA antibodies [12].

Otherwise, the immune response to CMV infection is complicated and complex, grouping various innate and adaptive immune responses (including antibody-mediated or humoral responses and responses mediated by cells or cellular responses). The immune system of graft recipients recognizes CMV and first triggers an innate response by producing type I IFN and inflammatory cytokines that initiate cellular and humoral responses that will be essential during this virus’ early viremic phase of infection.

In transplant recipients with immunosuppressants, we have a brake and a limited attenuation of the primary responses, both innate and adaptive, slowing down the response of NKs cells and causing a continuous decrease in immunoglobulin levels and the levels of specific antibodies against CMV [13]. BCMA levels may, in this case, be essential to modulating this production of CMV-specific antibodies. Likewise, the response of CMV-specific T cells is also slowed down, the scope and duration of which can predict the risk of progression to CMV viremia and favor the reactivation of the virus post transplant.

Indeed, the quantification of anti-CMV antibody titers and lower anti-PPS antibody concentrations were independent predictors of CMV disease and bacterial infections, respectively. Higher pre-transplant BAFF levels were also a risk factor for acute cellular rejection [14].

In this sense, in a multicenter study [15], these same authors found that 10% of the patients had CMV disease after lung transplantation. Post transplantation, IgG hypogammaglobulinemia was associated with an increased risk of CMV disease and fungal infection, and higher pre-transplantation BAFF levels were associated with a higher rate of development of severe bacterial infections and acute cellular rejection. This, i.e., the relationship between CMV status or reactivation, could be modulated by a differential BAFF molecule system expression.

We also examined the levels of BAFF system transcripts in KT recipients and their associations with B cell subtypes and CMV reactivation both before and after the transplant in this study.

First, we investigate the dynamics of CMV reactivation and the gene expression of the BAFF system molecules during the post-transplantation period. Our findings demonstrate that, aside from the levels of BCMA transcripts, which are elevated in these graft recipients experiencing CMV reactivation, the levels of BAFF, R-BAFF, and TACI transcripts after transplantation are sufficient and stable and do not differ from those observed at pre transplantation.

No information is available regarding the dynamics of BAFF system transcripts in kidney transplantation, with a deficient presence in the reported literature. An article in this vein finds that, in contrast to healthy controls and dialysis patients, recipients’ BAFF mRNA levels increased from the first year after transplantation, according to research by Xu et al. [12].

Contrarily, immune system cells, including macrophages and dendritic cells, are the cytokine APRIL’s main producers [16]. Immune activation following transplantation is well known to come from cellular damage brought on by ischemia episodes or surgery, among other causes. APRIL expression may rise as a result of this intrinsic stimulation. Nonetheless, an additional study should back up this hypothesis [17].

According to our research on the soluble forms of the BAFF system [2,3], APRIL levels in serum fall off after transplantation. As a result, the rise in the expression of the hypothetical gene APRIL may also be a homeostatic mechanism for restoring basal levels from before the transplant.

The majority of studies have used soluble and non-transcribed BAFF, so it must be remembered that the findings might not exactly match the same assessed reality [18,19]. According to research by Thibault-Espitia et al., high amounts of R-BAFF transcripts and low BAFF transcripts are linked to a higher risk of long-term graft malfunction [20]. Higher levels of BAFF and BCMA gene expression have been linked in transcriptomics studies to biopsy samples that had been rejected [21]. Although BAFF dysregulation has been associated with antibody-mediated immune responses, this cytokine can stimulate T cell activation and promote differentiation toward the Th1 phenotype, leading to pro-inflammatory responses [22,23].

APRIL/TACI signaling is activated in plasmablasts and plasma cells, promoting its activation and survival and directly influencing humoral responses [24]. Studies in animal models also show that TACI activation facilitates IgG1 release and plasmablast differentiation of B cells [25].

In this sense, our study on B cell subtypes showed results with apparent borderline significance for plasmablasts and class-switched B cells, although this was not significant. More studies and a more extensive cohort will be necessary to clarify these facts.

According to recent research, BAFF is expressed outside of hematopoietic cells and can also be synthesized by the renal epithelium in response to specific stimuli [25]. The infiltration of B lineage cells, such as plasmablasts and plasma cells, into the graft during rejection would account for the rise in BCMA expression [26,27].

According to Thaunat et al. [27], local anti-HLA antibody production in tertiary lymphoid organs within the graft preserves the allo-specific humoral responses throughout chronic rejections. An increase in B cell differentiation into antibody-secreting cells could be indicated by increased BCMA level expression into kidney allografts.

There is evidence that CMV may be related to the development of chronic vascular disease in kidney transplants. The impact of the indirect effects of CMV in several renal compartments is the object of speculation, and it was impossible to associate CMV infection with a particular inducing factor.

Another interesting point is that CMV viremia could also influence long-term outcomes with different impacts among different donor ages [28]. We have analyzed this parameter and our median donor’s age was 60.9 years, but we did not find a relationship between CMV viremia and donor age in our series.

According to our findings, renal graft recipients with early CMV reactivation had up-regulated BCMA transcript levels at the pre-transplantation stage, and its transcript levels positively correlate with peak viremia (>10,000 copies/mL). Nevertheless, these differences were not observed at the soluble BAFF level. High expression of the BCMA receptor in patients with early CMV reactivation also correlated with elevated plasmablasts and B cell class-switched levels and viral load. These results imply, with a possible translation into routine clinical practice, that levels of BCMA transcripts may be clinically helpful in predicting CMV reactivation risk in kidney allograft recipients after transplantation and helpful in the eventual adoption and modification of treatment.

This article has several important limitations that should be noted. Further research, including an extensive cohort and more thorough follow-up, will be required to validate our results and prove a true connection between the BAFF system molecules, CMV reactivation, and kidney transplant outcome. The kind of induction therapy, the dosage of immunosuppression, and the influence of other infections on kidney recipients are potential factors that might impact transcript levels but have not been adequately investigated. With respect to the adopted induction regimens in this cohort, several patients had not received induction, which may be a confounding factor and this particular point could also be important in future studies. Another eventual limitation of our study is the low number of rejection episodes in our patients, although it should be taken into account the shorter follow-up period of this study. To end, another additional and possible limitation of our study could be the lack of measurement of the blood level of anti-CMV antibodies in the two groups of analyzed patients, which should be considered in future studies. Furthermore, there are no data for the correlation between BCMA and immunosuppressant drug levels, which should also be evaluated in future studies.

Finally, increasing the number of analyzed transplants is strictly necessary to corroborate these preliminary results. In order to observe the ultimate impact on graft function and alloantibody production, more extended follow-up periods are also required.

## 4. Materials and Methods

### 4.1. Study Design, Clinical Parameters, and Demographic Information

A total of 30 CMV-IgG seropositive KT recipients (KTR) at the University Clinic Hospital “Virgen de la Arrixaca” from the Southeast of Spain were included in this study.

Only those patients with kidney grafts in operation for at least one month post transplantation and who had complete clinical data were included in our study. Allograft loss was estimated as a return to dialysis.

All transplant patients had an estimated glomerular filtration rate (eGFR) and creatinine analysis (normal values between brackets): eGFR (>90 mL/min/1.73 m^2^) and creatine (0.7 to 1.2 mg/dL). The pre-transplant values for our patient cohort were as follows: a creatinine level of (2.92.1 mg/dL; mean SD) and an eGFR of less than 60 mL/min/1.73 m^2^ for more than three months, suggestive of chronic kidney disease as previously published [29]. In addition, and for more clinical information, the percentage of patients who later were transplanted with peritoneal dialysis was 16.7% (5/30), and hemodialysis was 83.3 (25/30). These data were very similar to other Spanish centers.

Through qPCR, CMV reactivation was tracked for the first three months following the transplant (CMV-R-gene, Biomeriux, Barcelona, Spain). Among KT recipients with at least one sample of more than 500 copies/mL, CMV reactivation was categorized as positive (CMV+ group, *n* = 12) and negative (CMV− group, *n* = 18). Regarding age, sex, HLA incompatibilities, and donor type, these chosen patients did not differ significantly.

Thymoglobulin was given to 14 (35%) patients as part of induction therapy, while basiliximab was given to five (12.5%) patients. None of the variables under analysis revealed any statistically significant differences.

All patients gave informed consent for inclusion before participating in the study. The study was conducted in accordance with the Declaration of Helsinki, and the protocol was approved by the Ethics Committee of HCUVA (PI15/01370).

### 4.2. Immunosuppressive Treatment and CMV Treatments

All patients underwent the same triple immunosuppressive regimen of prednisolone, oral tacrolimus (TR) (Prograf, Astellas, Ireland), and mycophenolatemofetil (MMF; CellCept, Roche, Switzerland) (Dacortin, Merck, Spain). The TR-based protocol was started at 0.10–0.15 mg/kg/day, and the dose was adjusted to maintain a trough level of FK in whole blood between 8 and 12 ng/mL during the first month postoperatively, between 7 and 10 ng/mL during the first 2–3 months following transplant, and between 5 and 8 ng/mL after that. As previously reported, MMF was started at a dose of 2000 mg/day and decreased to 1000–1500 mg/day during the first month following surgery, depending on the white blood cell count [29].

On the day of the transplant, the first day after the operation, and the third day after, methylprednisolone was given intravenously at doses of 500, 250, and 125 mg/day, respectively. On the fifth day following surgery, oral prednisolone was started at a dose of 20 mg, and within two to three months after the transplant, the dose was tapered to 5–10 mg/day. Depending on the immune risk before transplantation, some cases were treated with thymoglobulin- or basiliximab-based induction therapy.

All patients with CMV reactivation received CMV therapy with valganciclovir (Valcyte, Hoffmann-La Roche, Basel, Switzerland) at 900 mg daily for six months. We used oral valganciclovir when the recipient could achieve targeted tacrolimus level only with oral tacrolimus. One patient resisted treatment and was administered a second-line agent: foscarnet (Clinigen Healthcare Ltd., Madrid, Spain).

### 4.3. Kidney Rejection Diagnosis

A rise in serum creatinine of at least 20% above baseline serum creatinine and biopsy evidence of rejection were considered to be signs of acute cellular rejection of allografts (ACR) (specimens were evaluated by light microscopy and immunofluorescence staining with a marker of classical complement activation (C4d) and classified following the Banff classification as revised in 2017) [30]. A positive C4d staining in peritubular capillaries, simultaneous DSA presence, and distinguishable histopathological findings are necessary to diagnose acute antibody-mediated rejection (AMR) [31]. For the kidney, it was agreed that, as previously reported, an AMR diagnosis requires the co-existence of DSA, distinguishable histopathological findings, and the deposition of C4d in peritubular capillaries [31].

Mild acute cellular rejection was managed with pulse steroids (500 mg methylprednisolone boluses) and increased maintenance immunosuppression (Banff grade I). The other ACR patients received anti-thymoglobulin (ATG). ACR Banff grade I, steroid-insensitive grade II, and grade III, as well as antibody-mediated rejection (AMR), were additional classifications for AMR. AMR was additionally treated with pulse steroids and intravenous immunoglobulin (0.25 gr/kg for the final session and 1 gr/kg (maximum 140 g) divided into two doses accompanied by plasmapheresis) (3 sessions a day, every five days). As previously reported, we administered 500 mg of anti-CD20 (Rituximab, Roche pharmaceuticals) intravenously to the patients [18,32,33]. In our cohort, the number of rejection episodes in both groups, CMV+ (without ACR or AMR) and CMV− (three ACR and two AMR), was one episode, but it should be taken into account the shorter follow-up period of this study.

### 4.4. Design of the Study of Gene Expression of Molecules of the BAFF System

Upon venipuncture, peripheral blood samples anti-coagulated with EDTA were obtained from all patients for gene expression and flow cytometry and separated by lymphoprep (Bomburg, Germany), as previously published [2].

Using quantitative PCR (qPCR), the BAFF-R (TNFRSF13C), TACI (TNFRSF13B), and BCMA (TNFRSF17) gene levels were assessed in PBL-extracted RNA before and after KT.

**Figure 4:**
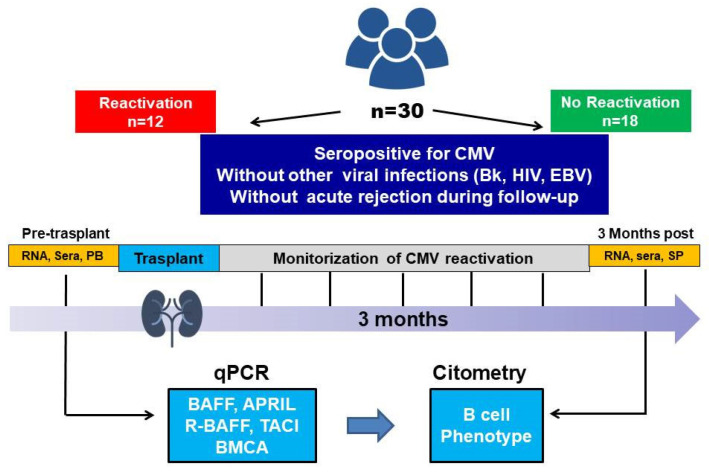
Detailed study design, flow, and sequential time steps of the work performed in this study. APRIL, A proliferation-inducing ligand; BAFF, B-cell-activating factor belonging to the TNF family; BMCA, B cell maturation Ag; Bk, virus BK; CMV, cytomegalovirus; EBV, Epstein–Barr virus; PB, peripheral blood; HIV, human immunodeficiency virus; qPCR, PCR quantitative; R-BAFF, receptor of BAFF; RNA, ribonucleic acid; TACI, transmembrane activator and CAML interactor.

Briefly, a prospective longitudinal study was conducted on the gene expression of molecules in the BAFF system. This was carried out using KTR samples collected before KT, and at three and six months after surgery (all the post-transplantation tests were strictly performed simultaneously to avoid eventual differences in other particular conditions and immunological status) (Figure 4). We used qPCR technique to measure the gene expression of the molecules in the BAFF system (BAFF, APRIL, R-BAFF, TACI, and BCMA). The results of the experimental study were used to establish a relationship between B cell subtypes, CMV reactivation, and BAFF system molecule transcript levels. A detailed study design, workflow, and sequential steps of the performed work are shown in Figure 4.

### 4.5. Total RNA Extraction

The Maxwell 16 miRNA Kit was used to isolate total RNA from PBLs by the manufacturer’s instructions (Promega, Cambridge, MA, USA).

Previously, PBL samples were lysed using Cell Lysis Solution Genomic Purification (Promega, MA). A NanoDrop2000 was used to measure the purity and quantity of the RNA (ThermoScientific, Waltham, MA, USA). Samples with 260/280 nm ratios of 2.0 to 2.2 were considered pure RNA. As previously reported, RNA integrity was evaluated using 1% agarose gel electrophoresis [18]. The purified RNA was then frozen at −60 °C until it was time to use it, as previously reported [2,3].

### 4.6. mRNA Reverse Transcription

mRNA was reverse-transcribed to complementary DNA (cDNA) using the RT2 First Strand Kit (Qiagen, Germantown, MD, USA). An amount of 1 µg of total RNA was treated with 2 µL of Buffer GE and 10 µL of RNase-free water for 5 min at 42 °C in order to eliminate genomic DNA. A final volume of 20 µL was obtained by combining the above mixture with 4 µL of BC3 buffer, 1 µL of Control P2, 2 µL of RE3 Reverse Transcriptase Mix, and RNase-free water. The mixture was incubated at 42 °C for 15 min. After incubation, the reverse transcription mixture was diluted to a final volume of 110 µL in water free of RNase. The cDNA samples were kept at −20 °C until they were used, as previously published [2,3].

### 4.7. Gene Expression of Molecules of the BAFF System

The gene expression of BAFF system molecules was carried out by qPCR. This was carried out using the following TaqMan-type hydrolysis probes (Applied Biosystem, Foster City, CA, USA): *HPRT1* (Hs99999909_m1), *CD19* (Hs00174333_m1), *TNFSF13B* (BAFF; Hs00198106_m1), *TNFSF13* (APRIL; Hs00601664_g1), *TNFRSF13C* (R-BAFF, Hs00606874_g1), *TNFRSF13B* (TACI, Hs00963364_m1), *TNFRSF17* (BCMA, Hs03045080_m1).

The expression of the HPRT1 gene served as an endogenous control for the normalization of BAFF and APRIL expression, whereas the expression of the CD19 gene was used to normalize the expression of R-BAFF, TACI, and BCMA genes. Using the 2-ΔΔCt method, the transcripts’ relative expression was calculated [34].

qPCR was performed using an ABI-7500 FastReal-Time PCR System (Applied Biosystem, Singapore). A PCR reaction mix containing 10 L of TaqMan Fast Advanced Master Mix (Applied Biosystem, Foster City, CA, USA), 7 L of nuclease-free water, and 1 L of the appropriate TaqMan probe was added for each qPCR reaction. The reaction mixture was then transferred to a 96-well plate, and 2 µL of cDNA or 2 µL nuclease-free water was added for patient samples or negative controls, respectively. The thermal cycler settings for the real-time PCR were 40 cycles of denaturalization for 3 s at 95 °C and primer union/extension for 30 s at 60 °C as previously published, 1 cycle for incubation UNG during 2 min at 50 °C, and 1 cycle for initial activation of Taq polymerase during 20 s at 95 °C [2,3,35,36].

### 4.8. Monitoring of B Cells Subpopulations by Flow Cytometry

Flow cytometry was used to monitor B lymphocyte (BL) subpopulations in PBL samples taken before surgery and three and six months after KT. Pre-KT samples were obtained and processed at the transplant center in less than 24 h. As in our earlier articles [18,19,37], T cells were also observed. PBL samples were labeled with monoclonal antibodies using the standard flow cytometry labeling technique.

Antibodies panel to monitor T and B cell subtypes by flow cytometry. Fluorocromes Tube 1: Lymphocytes B, Tube 2: Lymphocytes T Antibody (Clon) Manufacturer Antibody (Clon) Manufacturer FITC CD19 (HIB19) Becton Dickinson, BD Biosciences, CD19 (HIB19) CD4 (RPA-T4) BD Biosciences, PE IgM (G20-127) BD Biosciences, CD25 (2A3) BD Biosciences, PE-Cy7 CD27 (M-T271) BD Biosciences, CD45RO (UCHL1) BD Biosciences, APC CD38 (HB7) BD Biosciences, BD Biosciences APC-Cy7 CD24 (ML5) BD.

In summary, 50 µL of PBL was treated with 5 µL of each monoclonal antibody for 10 min in the dark. Samples were incubated with 3 mL of BD FACS Lysing Solution for 7 min at room temperature following the lysing stage (BD, Bioscience, San Jose, CA, USA), as previously published [18]. As stated in a previous paper, materials were lysed and centrifuged for five minutes at 1800 rpm [7,11]. After labeling, samples were acquired on a FACS Canto II flow cytometer (BD, San Jose, CA, USA). The results were analyzed and evaluated in the FACS Diva software (BD, San Jose, CA, USA). The absolute number of the different cell subpopulations may be calculated by comparing the relative abundances acquired by flow cytometry to the absolute lymphocyte count obtained using a Medtronic cell counter M16 (Boule Medical, Stockholm, Sweden). The phenotype of the lymphocyte subpopulations, gating strategies, and visual analysis techniques were used as previously described [33]. Panels from a collection of studies served as the foundation for the phenotype of the T and B lymphocyte subpopulations [35,37].

### 4.9. Statistical Analysis

For quantitative data, the results were presented as the mean, standard error of the mean (SEM), or percentages for categorical data. A comparison of categorical variables was performed using Fisher’s exact or X^2^ tests. The Kolmogorov–Smirnov test was used to determine whether the data were normal. The Mann–Whitney U test compared two groups using variables that did not account for normal distribution. The Kruskal–Wallis test and Dunn’s post hoc test with Bonferroni correction for multiple comparisons were used to compare three or more groups. Correlation analysis using the previously reported Spearman index was conducted [18,29].

The Wilcoxon non-parametric test for related samples was used to compare two related groups over time. The Friedman test compared three similar groups using Wilcoxon post hoc analysis.

The construction of ROC curves was used to assess the sensitivity and specificity of a biomarker. The Area Under the Curve (AUC) assessed the discriminating power. We utilized the Youden index to obtain the ideal cut-off value that maximized sensitivity and specificity.

The Benjamini–Hochberg or Bonferroni procedure also adjusted the *p*-value in multiple comparisons. For all statistical tests, *p*-values of 0.05, or p-corrected 0.05 in the event of multiple comparisons, were considered significant, as previously published [37,38].

According to previously published methods, the relative frequencies obtained from the cytometry analysis for each cellular subpopulation were normalized [33,37].

Statistical Package for the Social Sciences (SPSS, version 27, Chicago, IL, USA), GraphPad Prism (version 6, San Diego, CA, USA), and the R programming language was used to create the graphs and conduct the statistical analyses. The environment used for the R programming language was Integrated Development RStudio version 3.4.

## Figures and Tables

**Table 1:** Demographic data and clinical characteristics of the transplanted patients specifically included in the BAFF system gene expression, CMV, and B cell subpopulation study.

	CMV+ (*n* = 12)	CMV− (*n* = 18)	*p* ^a^
Age (years)	56.1 ± 1.59	56.0 ± 6.71	0.365
Gender (Male/Female)	7 (58.3)/5 (41.7)	10 (55.6)/8 (44.4)	1.000
HLA Mismatches ^b^	4.1 ± 0.17	4.5 ± 0.64	0.524
Live Donor (%)	2 (16.7)	1 (5.6)	0.338
Preformed anti-HLA antibodies (%)	1 (8.3)	6 (33.3)	1.000
Induction therapy (Tim/Bas)	8 (66.7)/2 (16.7)	11 (61.1)/5 (27.8)	0.386
Delayed graft function (%)	2 (16.7)	8 (44.4)	0.279
Type of rejection (Cellular/Humoral)	0 (0.0)	3 (16.7)/2 (11.1)	-
**Transplantation indications**	**CMV+** ***n* (%)**	**CMV−** ***n* (%)**	**Total** ***n* (%)**
Glomerulonephritis	4 (33.3)	5 (27.7)	9 (30.0)
Polycystic kidney disease	2 (16.7)	3 (16.7)	5 (16.7)
Type I diabetes mellitus	2 (16.7)	2 (11.1)	4 (13.3)
Chronic obstructive pyelonephritis	1 (8.3)	2 (11.1)	3 (10.0)
Unknown renal insufficiency	1 (8.3)	2 (11.1)	3 (10.0)
Lupic nephritis	1 (8.3)	1 (5.6)	2 (6.7)
Reflux nephropathy	1 (8.3)	1 (5.6)	2 (6.7)
IgA nephropathy	0 (0.0)	1 (5.6)	1 (3.4)
Atypical hemolytic uremic syndrome	0 (0.0)	1 (5.6)	1 (3.4)

Bas, Basiliximab; Tim, Thymoglobulin; NRA, No Acute Rejection; RA, Acute Rejection. The mean minus the standard error of the mean is how quantitative data are expressed (SEM). Quantitative data were expressed as the mean value ± SD. ^a^ Comparisons were made using Fisher’s exact test or X^2^ for qualitative variables and the non-parametric Mann–Whitney U test for quantitative variables. Values of *p* < 0.05 were considered significant. All patients with CMV+ were treated with anti-CMV therapy. ^b^ HLA-A, HLA-B, and HLA-DRB1 gene variations overall between donor and recipient.

## Data Availability

No new data were created or analyzed in this study. Data sharing is not applicable to this article.
